# Chronic Intermittent Hypoxia-Induced Vascular Dysfunction in Rats is Reverted by *N*-Acetylcysteine Supplementation and Arginase Inhibition

**DOI:** 10.3389/fphys.2018.00901

**Published:** 2018-07-24

**Authors:** Bernardo J. Krause, Paola Casanello, Ana C. Dias, Paulina Arias, Victoria Velarde, German A. Arenas, Marcelo D. Preite, Rodrigo Iturriaga

**Affiliations:** ^1^Division of Pediatrics, Faculty of Medicine, Pontificia Universidad Católica de Chile, Santiago, Chile; ^2^Division of Obstetrics & Gynecology, Faculty of Medicine, Pontificia Universidad Católica de Chile, Santiago, Chile; ^3^Laboratorio de Neurobiología, Facultad de Ciencias Biológicas, Pontificia Universidad Católica de Chile, Santiago, Chile; ^4^Departamento de Química Orgánica, Facultad de Química, Pontificia Universidad Católica de Chile, Santiago, Chile

**Keywords:** arginase, chronic intermittent hypoxia, endothelial dysfunction, nitric oxide, oxidative stress, vascular reactivity

## Abstract

Chronic intermittent hypoxia (CIH), the main attribute of obstructive sleep apnea (OSA), produces oxidative stress, endothelial dysfunction, and hypertension. Nitric oxide (NO) plays a critical role in controlling the vasomotor tone. The NO level depends on the L-arginine level, which can be reduced by arginase enzymatic activity, and its reaction with the superoxide radical to produce peroxynitrite. Accordingly, we hypothesized whether a combination of an arginase inhibitor and an antioxidant may restore the endothelial function and reduced arterial blood pressure (BP) in CIH-induced hypertensive rats. Male Sprague-Dawley rats 200 g were exposed either to CIH (5% O_2_, 12 times/h 8 h/day) or sham condition for 35 days. BP was continuously measured by radio-telemetry in conscious animals. After 14 days, rats were treated with 2(*S*)-amino-6-boronohexanoic acid (ABH 400 μg/kg day, osmotic pump), *N*-acetylcysteine (NAC 100 mg/kg day, drinking water), or the combination of both drugs until day 35. At the end of the experiments, external carotid and femoral arteries were isolated to determine vasoactive contractile responses induced by KCL and acetylcholine (ACh) with wire-myography. CIH-induced hypertension (~8 mmHg) was reverted by ABH, NAC, and ABH/NAC administration. Carotid arteries from CIH-treated rats showed higher contraction induced by KCl (3.4 ± 0.4 vs. 2.4 ± 0.2 N/m^2^) and diminished vasorelaxation elicits by ACh compared to sham rats (12.8 ± 1.5 vs. 30.5 ± 4.6%). ABH reverted the increased contraction (2.5 ± 0.2 N/m^2^) and the reduced vasorelaxation induced by ACh in carotid arteries from CIH-rats (38.1 ± 4.9%). However, NAC failed to revert the enhanced vasocontraction (3.9 ± 0.6 N/m^2^) induced by KCl and the diminished ACh-induced vasorelaxation in carotid arteries (10.7 ± 0.8%). Femoral arteries from CIH rats showed an increased contractile response, an effect partially reverted by ABH, but completely reverted by NAC and ABH/NAC. The impaired endothelial-dependent relaxation in femoral arteries from CIH rats was reverted by ABH and ABH/NAC. In addition, ABH/NAC at high doses had no effect on liver and kidney gross morphology and biochemical parameters. Thus, although ABH, and NAC alone and the combination of ABH/NAC were able to normalize the elevated BP, only the combined treatment of ABH/NAC normalized the vascular reactivity and the systemic oxidative stress in CIH-treated rats.

## Introduction

Obstructive sleep apnea (OSA), a growing breathing disorder featured by cyclic episodes of partial or total airflow occlusions during sleep, is considered an independent risk factor for develop systemic hypertension and is linked with stroke, coronary artery disease, and pulmonary hypertension (McNicholas et al., 2007; Dempsey et al., 2010). The airflow occlusion produces hypoxia and hypercapnia, which in turn stimulates the carotid body (CB) causing sympathetic, hypertensive, and ventilatory responses. Among these alterations, chronic intermittent hypoxia (CIH) is considered the main factor to develop systemic hypertension (Lavie, 2003; Kheirandish-Gozal and Gozal, 2008; Dempsey et al., 2010). Pre-clinical models of rodents exposed to CIH, which mimics most of the pathologic features of OSA including hypoxemia and hypertension, are used to study the mechanisms involved in cardiovascular and autonomic alterations induced by OSA (Fletcher, 2000; Del Rio et al., 2010; Prabhakar and Kumar, 2010; Iturriaga et al., 2017). Although the link between OSA and hypertension is well established, the mechanisms underlying the onset and progression of the arterial blood pressure (BP) elevation are not well-known. It has been proposed that CIH produces oxidative stress, inflammation, and sympathetic overflow, endothelial dysfunction, and hypertension (Lévy et al., 2008; Garvey et al., 2009; Iturriaga et al., 2009). In addition, new evidences suggest that the CB is involved in generation of autonomic and cardiovascular and ventilatory alterations elicited by CIH (Iturriaga et al., 2014, 2017; Prabhakar et al., 2015). The cycles of hypoxia-reoxygenation produce oxidative stress in the CB and enhance its chemosensory responsiveness to hypoxia. The enhanced CB chemosensory drive leads to sympathetic hyperactivation of the sympatho-adrenal axis and the renin-angiotensin system (Fletcher, 2000; Iturriaga et al., 2009; Prabhakar et al., 2012).

OSA patients show endothelial dysfunction with reduced vasodilatation to ACh, and vascular remodeling characterized by increased intima-media thickness (Ip et al., 2004; Patt et al., 2010) Similarly, CIH diminish the vasodilatation induced by ACh in rats (Tahawi et al., 2001; Dopp et al., 2011). CIH produces structural changes in the rat skeletal muscle resistance arteries in the first 14 days of CIH (Philippi et al., 2010). We previously found evidence that endothelial dysfunction in CIH-induced hypertension, may result from an imbalance in the ratio of arginase-1 to eNOS expression, vascular remodeling and increased contractile capacity (Krause et al., 2015). Indeed, we found that *ex vivo* acute arginase inhibition in carotid arteries of CIH-treated rats reverted the impaired ACh-induced relaxation, an effect completely blocked by the NO-synthase inhibitors NG-nitro-L-arginine (L-NA). In addition, we found that arginase-1 protein level was increased, whereas eNOS levels decreased in CIH-derived arteries (Krause et al., 2015). Thus, it is plausible that the reduction of the oxidative stress and inhibition of the arginase enzymatic activity and may revert the vascular dysfunction and hypertension associated with CIH. It is well-known that NO levels play a critical role in vasomotor regulation, depending on L-arginine availability, which can be reduced in conditions where high arginase expression and activity have been evidenced (Demougeot et al., 2005; Bagnost et al., 2010; Krause et al., 2012; Cowburn et al., 2016). In addition, NO may react with the superoxide radical to produce peroxynitrite (ONOO^−^). Accordingly, we hypothesized whether the administration of an arginase inhibitor and a precursor of the potent antioxidant glutathione, *N*-acetylcysteine (NAC) (Rushworth and Megson, 2014; Lasram et al., 2015; Schmitt et al., 2015), may reduce the endothelial dysfunction and hypertension induced by CIH. Thus, we assessed the effects of the arginase inhibitor 2(*S*)-amino-6-boronohexanoic (ABH) and the antioxidant NAC on the elevated BP and endothelial dysfunction in carotid and femoral arteries, from CIH-induced hypertensive rats. Furthermore, since arginase inhibition could interfere with the urea pathway, the effects of high doses of ABH-NAC on renal and hepatic function on rats were assessed. In a separate series, we studied the effect of a large dose of ABH of 400 μg/kg day and NAC 400 mg /kg day on renal and hepatic function and histology in rats. Proteins, creatinine, and urea were determined in urine and creatinine, urea, glutamic-oxalacetic transaminase (GOT), glutamic-pyruvic transaminase (GPT), and lactate dehydrogenase (LDH) were measured in plasma.

## Materials and methods

### Animal care and ethics approval

This study was carried out in accordance with the recommendation of the Guide for the Care and Use of Laboratory Animals of the Bioethics Committee, CONICYT Chile. The experimental protocol for the animal was approved by the Bioethics Committee of the Faculty of Biological Sciences of the Pontificia Universidad Católica de Chile. For human samples the research was conducted in accordance with the Declaration of Helsinki and was approved by the Ethics Committee from the Faculty of Medicine of the Pontificia Universidad Católica de Chile (Protocol #11-247) as well as patient informed consent was obtained. Placentae were collected immediately after delivery from full-term normal normotensive, non-alcohol non-smoking, or drug consuming mothers, without any other obstetrical or medical problem.

### Animals and exposure to chronic intermittent hypoxia

Experiments were done on male Sprague-Dawley rats, weighing initially ~200 g. Rats were fed with standard rat chow diet *ad libitum*, and kept on a 12-h light/dark schedule (8:00 a.m.−8:00 p.m.). Rats were exposed to CIH for 35 days, as previously described (Iturriaga et al., 2010; Del Rio et al., 2016). Briefly, unrestrained, freely moving rats were housed in individual chambers. The CIH protocol consisted of hypoxic cycles of 5–6% inspired O_2_ for 20 s, followed by room air for 280 s, applied 12 times/h, 8 h/day. The chambers had a rear N_2_ inlet and a front air extractor, which enables the recovery to normoxia. A computerized system controlled the valve inlets and the alternating cycles of the extractors. During hypoxia, the extractors stopped for 30 s, and the rear solenoid valves allowed 100% N_2_ flow into the chambers. The O_2_ level in the chambers was continuously monitored with an oxygen analyzer (Teledyne AX 380, USA). The CO_2_ in the chamber was maintained low by continuous air extraction. In the Sham condition, rats were exposed to compressed air flow into chambers.

### Arterial blood pressure recordings

Arterial blood pressure (BP) was measured in conscious rats using radio-telemetry. Seven days before the beginning of the experiments, rats were anesthetized with 5% isoflurane and maintained with 2% isoflurane in 100% O_2_ during the surgical procedure. The tip of a cannula-coupled telemetry device (PC40A-40, Data Science International, USA) was introduced into the femoral artery (Del Rio et al., 2016). After surgery rats received a subcutaneous injection of ketoprofen (1%) and enrofloxacin (1%). The BP recording was started after 5 days of recovery. The raw data from each rat was continuously collected at a sample rate of 1 KHz, and average every 24 h with the ART Dataquest Platform (Data Science International, USA).

### Synthesis and functional studies of ABH

The 2(*S*)-amino-6-boronohexanoic acid (ABH) was synthesized by an optimization of a method previously reported by Vadon-Legoff et al. (2005), which was itself based in the previous work of Collet et al. (2000). The synthesis of ABH consisted of the preparation of the Gly-Ni-BPB complex, which was obtained by reaction of glycine (Gly), nickel(II) chloride, and 2[N-(N′-benzylprolyl)amino]benzophenone (BPB) in the presence of excess KOH in methanol. The Gly-Ni-BPB complex was treated with a base at low temperature and enantioselectively alkylated with the corresponding catechol-protected boronated bromide. The alkylated complex was then hydrolyzed with aqueous acid and purified by ion-exchange chromatography. The effectiveness of synthesized ABH was determined by measuring its ability to inhibit arginase activity, as described previously for endothelial cells (Krause et al., 2012). Briefly, basal arginase activity was determined in the presence of the synthesized inhibitor ABH and, compared with the effect of the commercial inhibitor of arginase, S-(2-boronoethyl)-L-cysteine (BEC. Sigma, USA) in protein extracts obtained from total human placental homogenates (i.e., vascular, syncytium and connective tissue) obtained with lysis buffer [1 mmol/l pepstatine A, 1 mmol/l leupeptine, 200 mmol/l phenylmethylsulfonyl fluoride (PMSF), 50 mmol/l Tris-HCl (pH 7.5), 0.2% Triton X-100] sonicated (20 pulses, 150 W, 3 min). The production of urea was measured using 70 μg of placental homogenate protein in 100 μl of reaction incubated at 37°C for 1 h in the presence of L-arginine (50 mM) and the cofactor Mn2+ (5 Mm), and the presence or absence of BEC (100 μM) or, increasing concentrations of synthesized ABH (10–1,000 μM). The catalysis was stopped by adding 4 volumes of acid solution (H2SO4: H3PO4: H2O = 1: 3: 7), and the urea formed was determined by adding 25 μl of α-isonitrosopropiophenone (9% ISPF in absolute ethanol) to the assay and heating the mixture (100°C for 45 min). The concentration of urea was measured by spectrophotometry (OD 450 nm) in an automatic plate reader based on results obtained from a urea calibration curve. We found that synthesized ABH produced a concentration-dependent inhibition of the arginase activity and that it reached levels comparable to those observed in the presence of the commercial inhibitor BEC. The Ki for ABH was 4.78 ± 1.76 μM and the solubility in water 125 g/L or 0.7 mol/L.

### Experimental procedure and drug administration

The antioxidant *N*-acetylcysteine (NAC, Sigma USA) was administered through drinking tap water (400 mg/kg day) from the day 14 of CIH exposure until the end of the experimental protocol. The NAC solution was prepared daily and preserved in dark condition to avoid oxidation. ABH was administrated with osmotic pumps (2ML4, Alzet Scientific Products, USA). Rats were anesthetized with isoflurane in O_2_, and the osmotic pumps were implanted subcutaneously on the back. Pumps deliver ABH in saline solution at a rate of 400 μg/kg/ day. Animals were implanted with the same osmotic pumps (Alzet Scientific Products, USA) filled with saline solution. Rats were out of the hypoxic chambers for 2–3 h for the surgical implant of the osmotic pumps. After surgical procedures, rats were treated with enrofloxacin and ketoprofen as mentioned before.

### Wire myography

At the end of the experiments, external carotid and femoral arteries were surgically removed from rats anesthetized with sodium pentobarbitone (50 mg/kg ip) and placed in ice-cold PBS. Vessel segments (~2 mm) of external carotid arteries and second/third-order femoral arteries were mounted on a wire-myograph (model 610A; Danish Myo Technology A/S, Denmark) and contractile responses were studied as previously described (Krause et al., 2015). Animals were euthanized with a higher dose of sodium pentobarbitone (200 mg/kg ip). The internal diameter of vessels was defined by determining the stretch condition at which the maximal contractile response to KCl was obtained (Delaey et al., 2002). Indeed, the internal diameter of the vessels for wire myography was established by determining the opening length (or stretching condition) at which the vessel presents its maximal contractile response (Mulvany and Aalkjaer, 1990). This *ex vivo* method has been shown to accurately represent the *in vivo* internal arterial perimeter in different models (Mulvany and Aalkjaer, 1990; Delaey et al., 2002). Likewise, through this methodology, a direct correlation of the *ex vivo* contractile response with the biomechanical and structural properties of different blood vessels has been observed (Cañas et al., 2017). Isometric force in response to cumulative concentrations of KCl (16–125 mM) was assayed to determine the maximal contractile force. Similarly, isometric force in response to acetylcholine (ACh, 10^−8^-10^−5^ mol/L) in pre-constricted vessels with 37.5 mmol/L KCl were determined. Responses were expressed as percentage of the maximal contractile effect induced by KCl at 40.8 mmol/L (%Kmax). Concentration-response curves were fit with the GraphPad Prism version 5.00 (CA, USA) to obtain the maximal effect and potency (pD_2_ = −log EC_50_).

### Measurement of systemic oxidative stress

Plasmatic oxidative stress was measured with the TBARS assay (Cat N° 10009055, Cayman, USA) according to the protocol provided by the supplier. The concentration of thiobarbituric acid-reactive species were expressed as malondialdehyde (MDA) μmol/L. Blood samples were collected from the common carotid artery and placed in heparinized ice-cold microcentrifuge tubes after 1–2 h of the last intermittent hypoxic cycle. Plasma was separated by centrifugation and stored at −80°C.

### Biosafety of NAC and ABH

Since arginase inhibition may affect the urea pathway, we evaluated whether high doses of ABH and NAC could have a hepatotoxic or nephrotoxic effect. In a separate series, two independent groups were used: one group without treatment (*n* = 4) and one treated with the combination of ABH and NAC (*n* = 8). Under isoflurane anesthesia, osmotic pumps were implanted subcutaneously (2ML4, Alzet Scientific Products USA). In the untreated animals, pumps were filled with saline solution. In the rats treated with ABH/NAC, the same osmotic pumps with ABH dissolved in saline solution were installed. Each rat received a dose of ABH of 400 μg/kg day and NAC 400 mg /kg day. Rats were kept for 28 days, and placed in metabolic cages to collect urine. Blood was collected from the ocular orbital sinus under isoflurane 5% in O_2_ anesthesia. Proteins, creatinine, and urea were determined in urine and creatinine, urea, lactate dehydrogenase (LDH), glutamic-pyruvic transaminase (GPT), and glutamic-oxalacetic transaminase (GOT) were measured in plasma.

In a separate experimental series of rats (*n* = 6) without treatment and a group of 6 rats treated with the combination ABH/NAC, creatinine clearance was measured. The collected urine was measured to determine the 24 h volume and then centrifuged at 3,000 rpm for 10 min at 4°C to discard any precipitate. Under anesthesia with isoflurane, blood was obtained from the left ventricle. Blood was collected in heparinized tubes, centrifuged at 3,000 rpm to obtain plasma. Creatinine was measured in both plasma and urine using the Creatinine Liquicolor kit from human (Wiesbaden, Germany), which evaluates the kinetics of the reaction. Briefly, plasma and urine samples (diluted 1:50) were mixed with a solution of picric acid and alkaline buffer (NaOH/bicarbonate). Immediately the samples were transferred to a reading cell and their absorbance was measured at 492 nm at 30 s and at 2 min after the start of the reaction. With these values and using a creatinine standard, the concentration of creatinine present in each sample was determined.

### Histological staining and examination

Anesthetized rats were perfused intracardially with phosphate buffer saline (PBS; pH 7.4) for 10 min, followed by buffered paraformaldehyde (PFA 4%, Sigma, St Louis, USA) for 10 min. The saline and PFA solutions were perfused at constant pressure of 95 mmHg. Pieces of the carotid external artery (3–4 mm length) were collected 2 mm rostral from the carotid sinus and post-fixed by immersion in buffered-PFA 4% for 12 h at 4°C. Carotid arteries were dehydrated in graded ethanol solutions followed by xylol, included in paraffin, sectioned at 5 μm and mounted on silanized slides. The vessels were stained with hematoxylin and eosin and photomicrographs were taken with an Olympus CX 31 microscope with a CCD camera (Olympus Corp, Japan). The internal diameter (ID) was measured from fixed tissues with the ImageJ software (NIH, USA).

Liver and kidney samples obtained from euthanized rats were fixed with 4% paraformaldehyde (Sigma, St Louis, USA), dehydrated and paraffin-embedded. Transverse sections (5 μM) were stained with Harris hematoxylin (5 min) and eosin (30 s) and mounted with Entellan (Merck, Whitehouse Station, NJ, USA). Microphotographs were obtained at 40x and 100x with and Olympus CX31 microscope (Olympus Corporation, Tokyo, Japan).

### Statistical data analysis

Data were expressed as mean ± SEM. For BP recordings, the average daily MABP for each animal were grouped and compared through one-way ANOVA followed by Dunnet' multiple comparison test among the treatment groups. Comparisons between two groups were performed with the U Mann-Whitney test, and differences between more groups were assessed with one or two-way ANOVA tests, followed by Dunnet *post-hoc* comparisons. Significance was accepted to *p* < 0.05.

## Results

### Effects of ABH and NAC on the arterial blood pressure in CIH-treated rats

**Figure 1:**
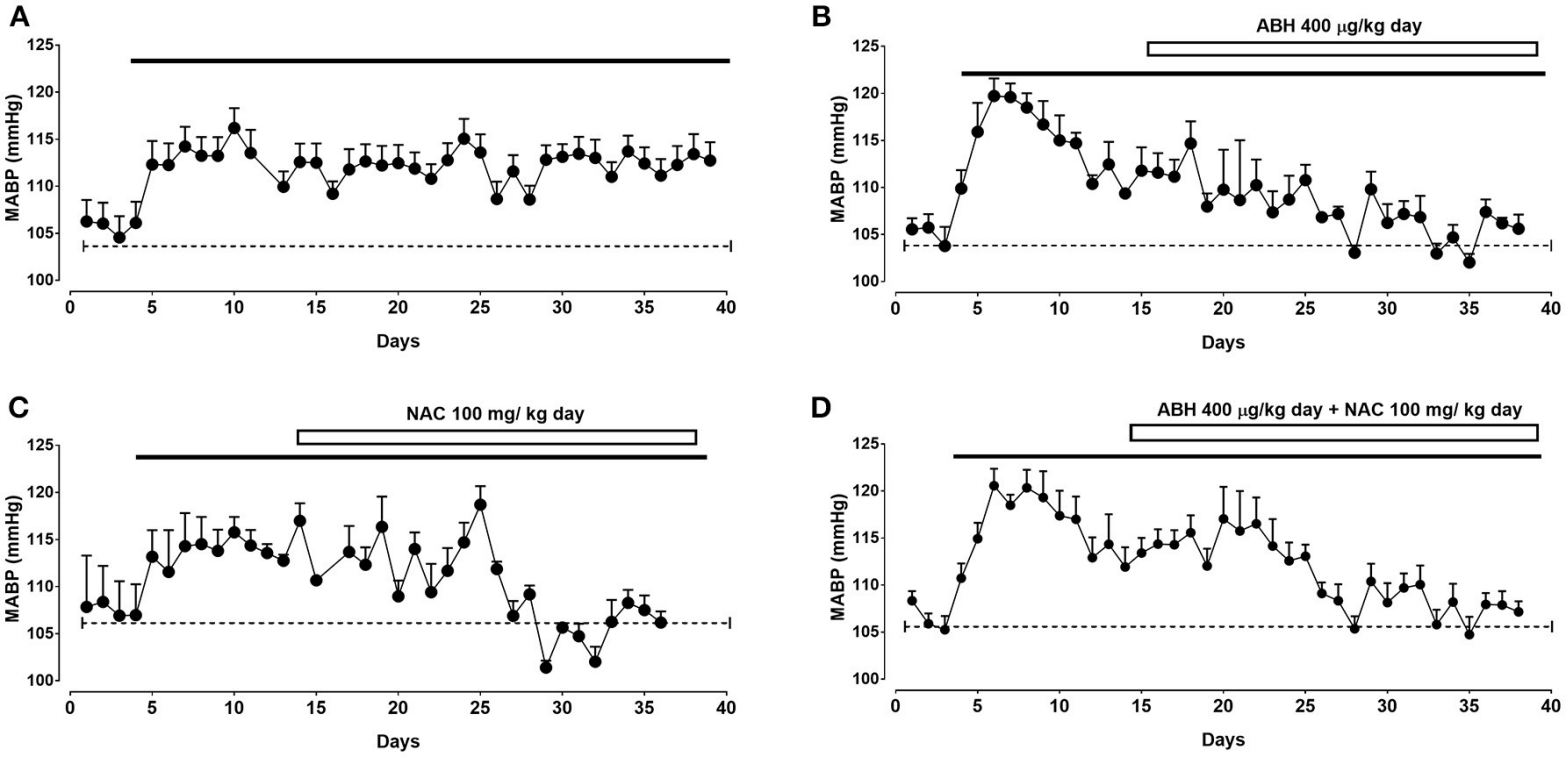
Effects of ABH and NAC on CIH-induced hypertension. Mean arterial blood pressure (MABP) determined by telemetry in rats submitted to CIH. After 14 days rats were maintained in CIH without treatment (**A**, *n* = 8) or receiving the arginase inhibitor ABH (**B**, *n* = 8), the glutathione precursor NAC (**C**, *n* = 6), or the combination of both ABH/NAC (**D**, *n* = 8).

Rats submitted to CIH showed a sustained increase in mean arterial blood pressure (MABP) of ~8 mmHg after 3–4 days of exposure (Figure 1 and Table 1). To demonstrate the role of arginase activity and oxidative stress in this increased arterial BP, rats exposed to CIH were treated with the arginase inhibitor ABH and/or NAC from day 14 of CIH. ABH and NAC treatment progressively reduced MABP in CIH-rats reaching basal values (Figures 1B,C). Similarly, simultaneous administration of ABH and NAC restored the normal MABP levels in CIH-treated rats (Figure 1D).

**Table 1:** Effects of ABH, NAC, and ABH/NAC on arterial blood pressure.

	**MABP baseline (mm Hg)**	**MABP 7–14 days (mm Hg)**	**MABP 28–35 days (mm Hg)**
SHAM	106.0 ± 1.4	107.4 ± 1.5	107.1 ± 1.1
CIH	105.8 ± 0.4	112.4 ± 0.1^*^	112.6 ± 0.4^*^
CIH+ABH	105.0 ± 0.6	112.9 ± 0.6^*^	105.1 ± 0.7
CIH+NAC	107.5 ± 0.4	114.0 ± 0.3^*^	105.9 ± 0.9
CIH+ABH/NAC	106.5 ± 1.4	116.7 ± 1.4^*^	107.1 ± 0.8

*Mean ± SEM*.

* *P < 0.05 vs. baseline. One-way ANOVA followed by Dunnet (n = 6–8 rats per group)*.

### Effect of ABH and NAC on the contractile responses in arteries from CIH-treated rats

**Figure 2:**
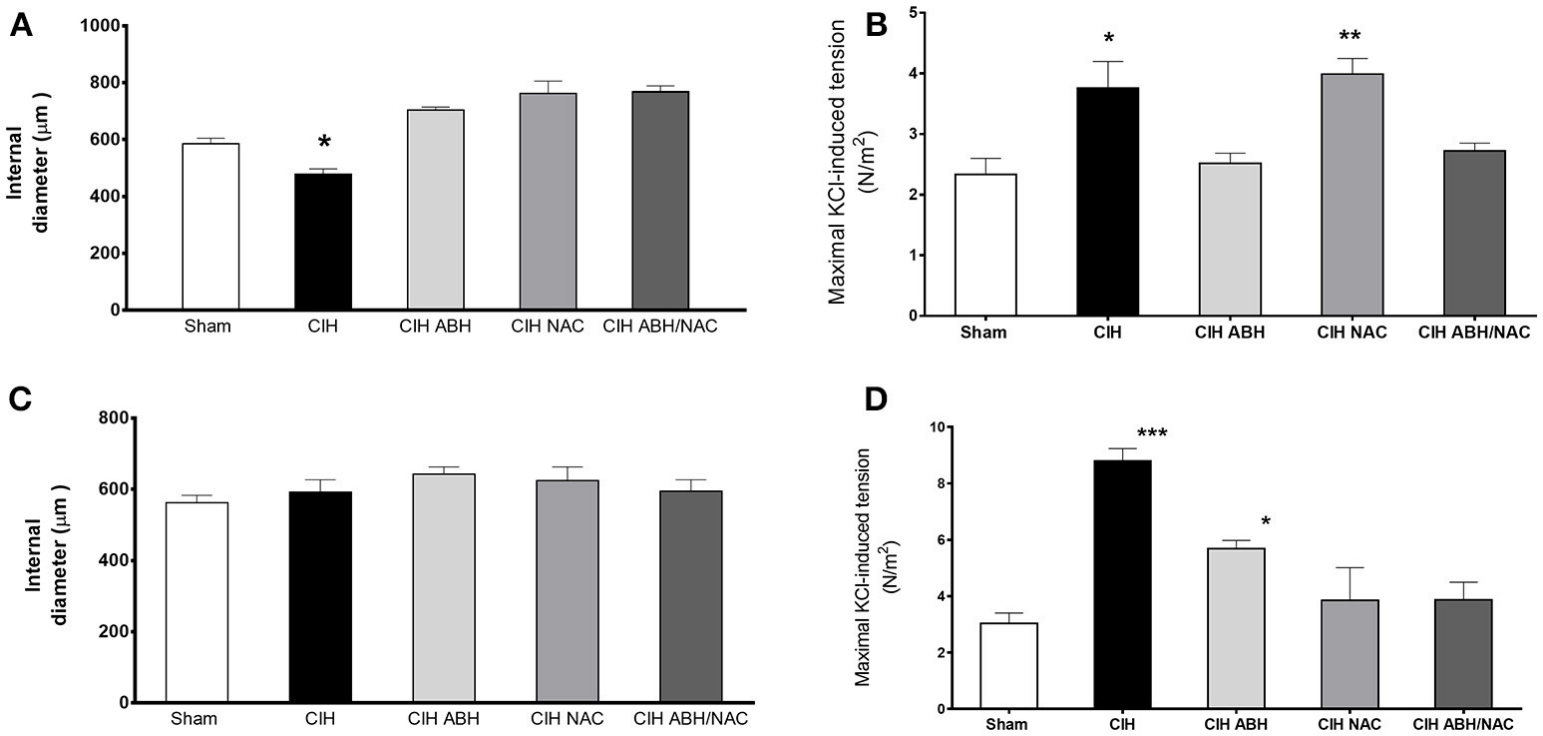
Effects of ABH and NAC on the CIH-induced vascular remodeling. Internal diameter measured by histology in fixed tissues **(A,C)** and maximal contractile response to KCl **(B,D)** of carotid **(A,B)** and femoral **(C,D)** arteries from sham (open bars, *n* = 10), CIH (solid bars, *n* = 10), CIH treated with ABH (light gray bars, *n* = 5), CIH treated with NAC (gray bars, *n* = 5), and CIH treated with ABH, and NAC (dark gray bars, *n* = 5) rats. Values expressed as mean ± SEM, *p < 0.05, **p < 0.01, ***p < 0.001 vs. sham, ANOVA.

To determine the effects of ABH and NAC on vascular reactivity in CIH-rats, the vasoactive response to KCl of carotid and femoral artery segments were assayed using wire myography. Carotid arteries from CIH-rats showed a significant decreased internal diameter measured in histological sections of fixed tissues, whilst the treatment with ABH, NAC and their combination ABH/NAC prevented the reduction in internal diameter (Figure 2A). Compared with sham, maximal KCl-induced constriction was increased in CIH and CIH NAC groups, while this effect was reverted by ABH, as well as the combination of ABH/NAC (Figure 2B). There were no significant changes in the internal diameter of femoral arteries in the groups studied compared with sham rats (Figure 2C). However, maximal KCl-induced constriction was increased in CIH. The latter effect was partially reverted by ABH treatment and completely prevented by NAC, as well as ABH/NAC (Figure 2D).

### Effect of ABH and NAC on the NOS-dependent relaxation in arteries from CIH-treated rats

**Figure 3:**
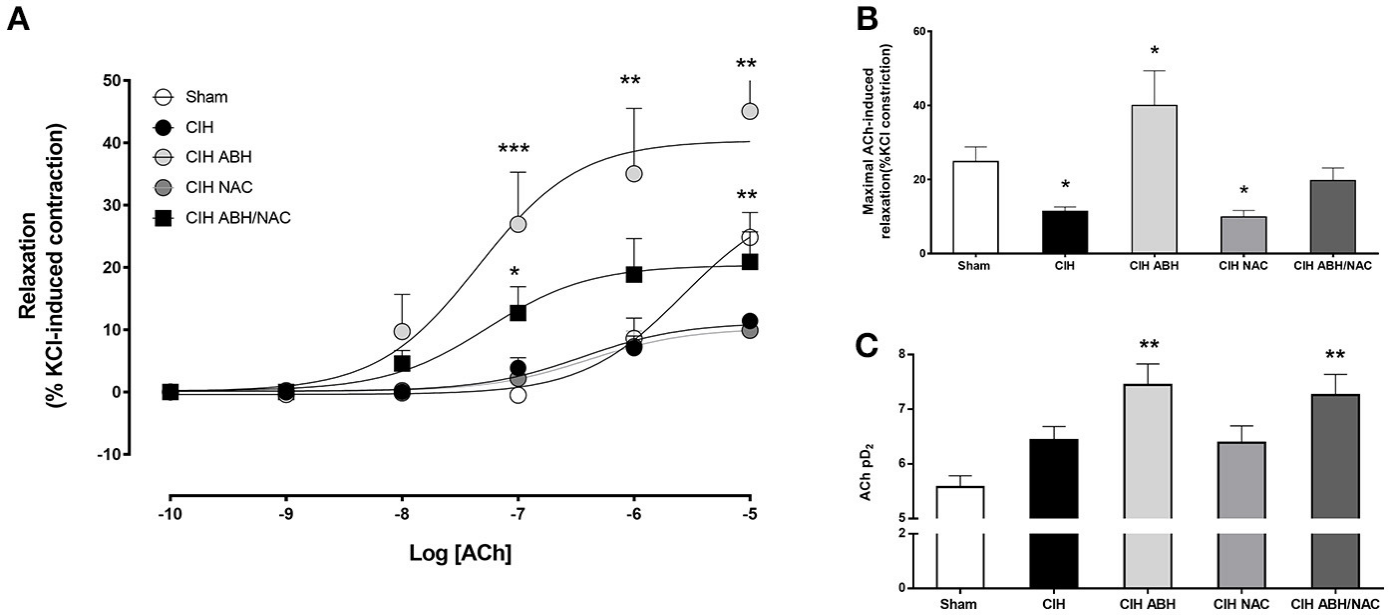
Endothelial-dependent relaxation in carotid arteries from CIH-rats. **(A)** Concentration-dependent relaxation curves in response to acetylcholine of carotid arteries from sham (open circles, *n* = 6), CIH (solid circles, *n* = 6), CIH treated with ABH (light gray circles, *n* = 6), CIH treated with NAC (gray circles, *n* = 6) and CIH treated with ABH, and NAC (dark gray square, *n* = 6) rats. Maximal acetylcholine-induced response **(B)** and pharmacological potency (pD_2_, i.e., sensitivity) **(C)** from sham (open bars), CIH (solid bars), CIH treated with ABH (light gray bars), CIH treated with NAC (gray bars), and CIH treated with ABH, and NAC (dark gray bars) rats were derived from **(A)**. Values expressed as mean ± SEM, *p < 0.05, **p < 0.01, ***p < 0.001 vs. sham, ANOVA.

**Figure 4:**
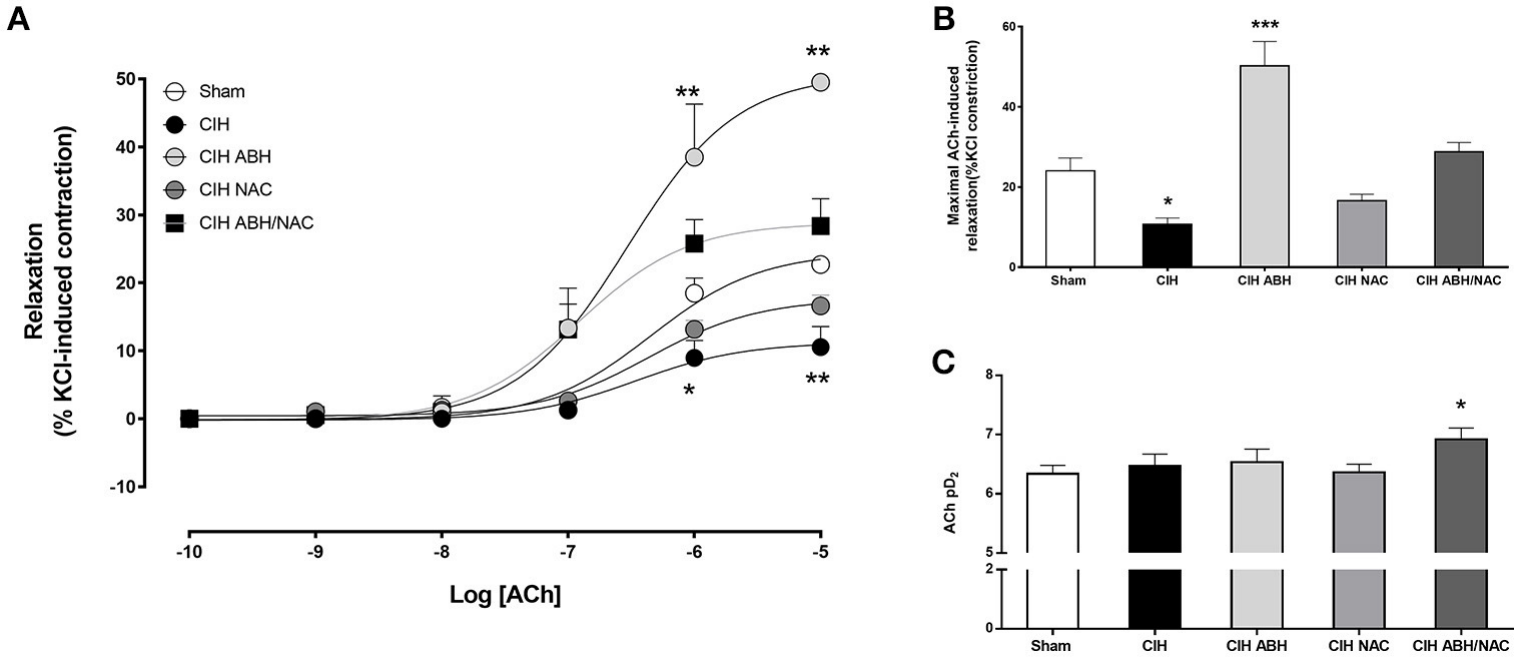
Endothelial-dependent relaxation in femoral arteries from CIH-rats. **(A)** Concentration-dependent relaxation curves in response to acetylcholine of femoral arteries from sham (open circles, *n* = 6), CIH (solid circles, *n* = 6), CIH treated with ABH (light gray circles, *n* = 6), CIH treated with NAC (gray circles, *n* = 6) and CIH treated with ABH, and NAC (dark gray square, *n* = 6) rats. Maximal acetylcholine-induced response **(B)** and pharmacological potency (pD_2_, i.e., sensitivity) **(C)** from sham (open bars), CIH (solid bars), CIH treated with ABH (light gray bars), CIH treated with NAC (gray bars), and CIH treated with ABH, and NAC (dark gray bars) rats were derived from **(A)**. Values expressed as mean ± SEM, *p < 0.05, **p < 0.01, ***p < 0.001 vs. sham, ANOVA.

Carotid arteries from rats exposed to CIH showed a decrease in the concentration-dependent relaxation (Figure 3A) and maximal response (Figure 3B) to ACh compared to the sham group, and this effect was reverted by the combination of ABH/NAC. Similarly, treatment with NAC did not improve the response to ACh in CIH-rats, whilst ABH treated rats showed a significant increase in the maximal response compared to sham. Sensitivity (pD_2_) to ACh was comparable between sham, CIH and CIH- NAC groups, but substantially increased (>1 Log unit) in CIH rats treated with ABH and ABH in combination with NAC (Figure 3C). Femoral arteries from rats exposed to CIH showed a decrease in the concentration-dependent relaxation (Figure 4A) and maximal response (Figure 4B) to ACh related to sham, effect reverted by NAC and ABH/NAC. CIH-ABH-treated rats showed a ~2-fold increase in the maximal response to ACh relative to the sham. Conversely, sensitivity to ACh was comparable between sham, CIH, CIH-ABH, and CIH-NAC groups, but substantially increased in CIH-rats treated with ABH in combination with NAC (Figure 4C).

### Effects of ABH and NAC on systemic oxidative stress in CIH-rats

**Figure 5:**
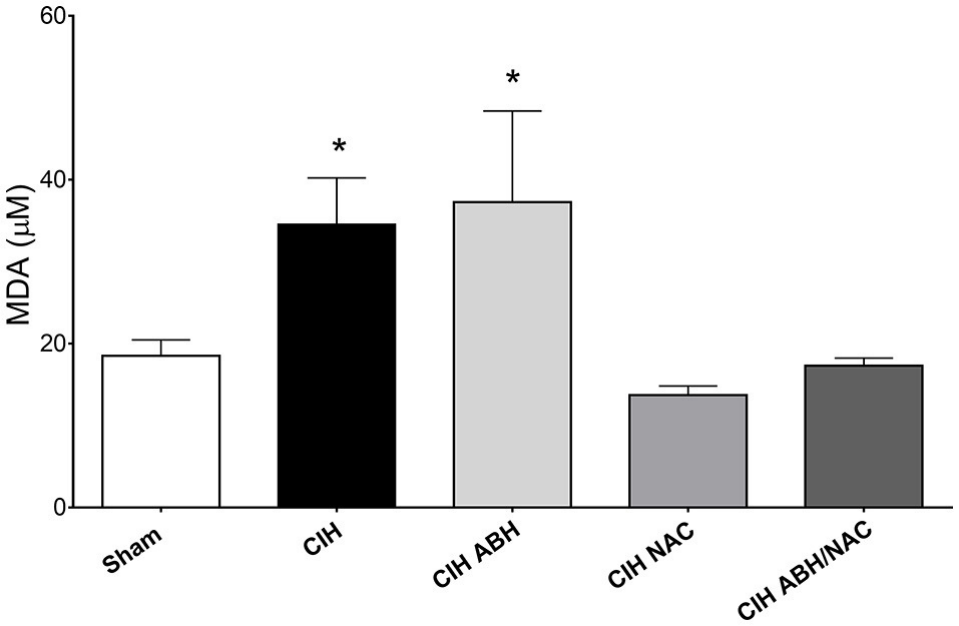
Circulating levels of oxidized lipids in CIH-rats. Plasma levels of the oxidative stress marker MDA in sham (open bars, *n* = 16), CIH (solid bars, *n* = 13), CIH treated with ABH (light gray bars, *n* = 7), CIH treated with NAC (gray bars, *n* = 5), and CIH treated with ABH, and NAC (dark gray bars, *n* = 10) rats. Values expressed as mean ± SEM, *p < 0.05 vs. sham, ANOVA.

Systemic oxidative stress in CIH-treated rats was evaluated by determining plasma levels of MDA as an index of lipids peroxidation after 35 days of CIH or sham condition. CIH-treated rats showed a substantial increase (~2-fold) in MDA levels compared with the sham group, an effect also observed in CIH-rats treated with ABH (Figure 5). On the contrary, CIH-rats treated with NAC, as well as with ABH/NAC, showed plasma levels of TBARS comparable to sham rats (Figure 5).

### Biosafety testing of ABH-NAC combination

**Figure 6:**
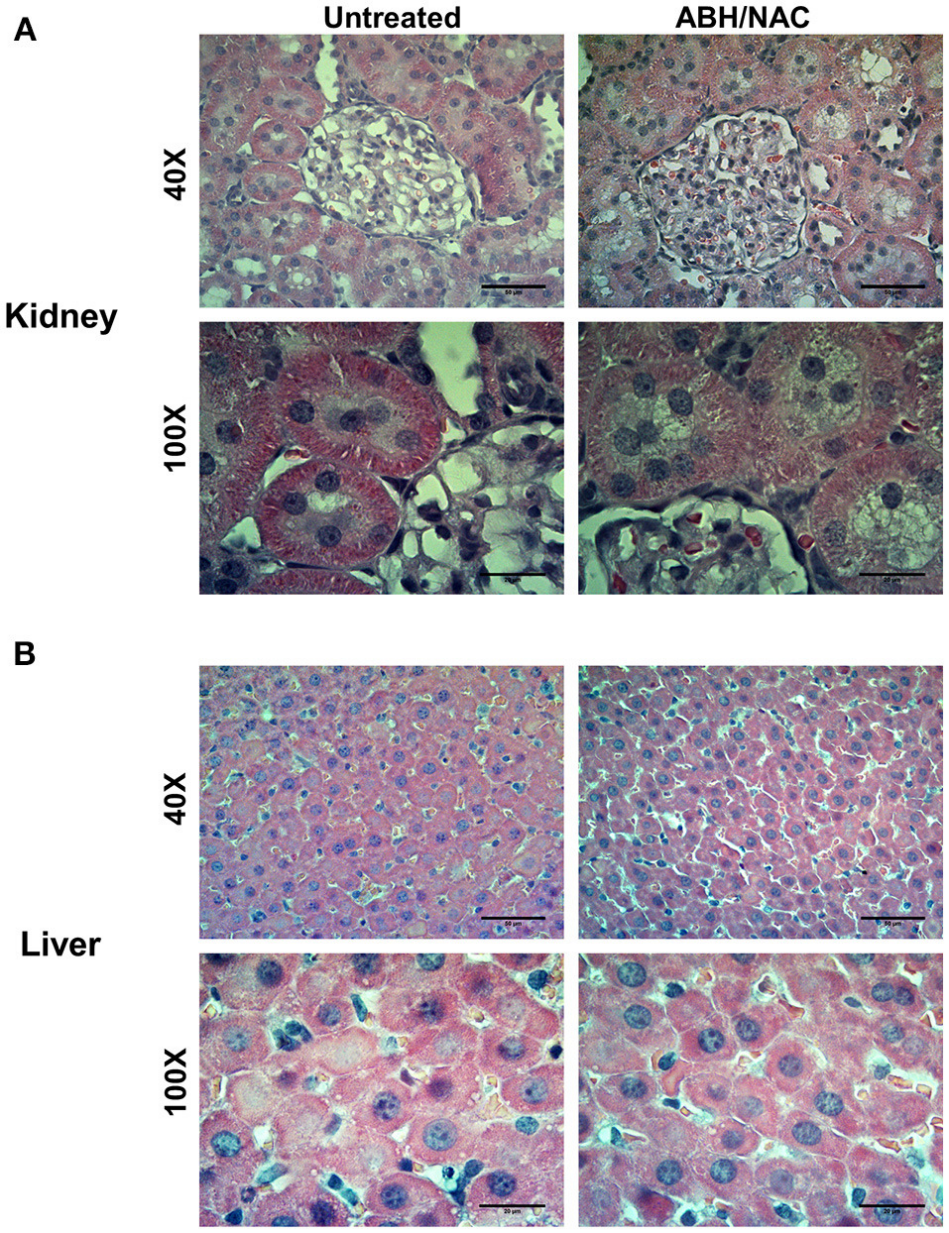
Effects of ABH/NAC treatment on kidney and liver morphology. Micrograph of transverse sections of kidney **(A)** and liver **(B)** samples stained with Harris hematoxylin and eosin from untreated and ABH/NAC treated rats.

Considering the effect of ABH/NAC combination on normalizing the BP and vascular reactivity in CIH rats, liver and kidney morphology as well as the renal function was evaluated in sham rats treated for 14 days with high doses of ABH (400 μg/kg day) and NAC (400 mg/kg day). Kidney (Figure 6A) and liver (Figure 6B) morphology were not altered by the treatment. Similarly, we did not found changes in plasma urea concentration and creatinine clearance between the rats with or without ABH/NAC treatment (Table 2).

**Table 2:** Effect of ABH/NAC on rats renal and hepatic variables.

	**Untreated**	**ABH/NAC**
Initial body weight (g)	199.8 ± 3.7	197.3 ± 1.9
Body weight at 28 days (g)	330.3 ± 4.2	298.3 ± 7.0^*^
Body weight as % initial (%)	165.3 ± 2.2	146.6 ± 3.6^*^
Plasma urea (mg/dL)	41.9 ± 3.4	48.0 ± 4.8
Urine urea (mg/24 h)	6.5 ± 0.4	5.4 ± 0.5
Proteins (mg/24 h)	10.7 ± 1.2	12.5 ± 1.3
Plasma creatinine (mg/dL)	0.56 ± 0.2	0.57 ± 0.3
Urine creatinine (mg/24 h)	972.0 ± 87.5	941.5 ± 57.1
Creatinine clearance (ml/min)	1.78 ± 0.3	1.76 ± 0.2
Lactate dehydrogenase LDH (U/L)	1111.0 ± 435.3	768.6 ± 188.9
Glutamic-oxalacetic transaminase GOT (U/L)	60.0 ± 4.5	61.0 ± 11.4
Glutamic-pyruvic transaminase GTP (U/L)	21.7 ± 1.1	16.5 ± 2.9

* *P < 0.05 U-Mann-Whitney treated vs. non-treated rats (Untreated Rats n = 4 and ABH-NAC rats = 8)*.

## Discussion

This study aimed to determine whether the administration of the arginase inhibitor ABH and the glutathione precursor NAC may revert the hypertension and endothelial dysfunction in rats exposed to CIH. The results showed that ABH and NAC, as well as the combination of both drugs, reverted the increase in BP induced by CIH. However, only the combination of ABH and NAC leads to a normalization of BP and endothelial function along with a reversion of vascular remodeling markers, as suggests the changes in contractile force and internal diameter. In addition, the combination of ABH and NAC at high doses had no effect on liver and kidney morphology and biochemical function.

Compelling evidence shows that, in humans, oxidative stress has a prominent role in the cardiovascular dysfunction induced by OSA, a fact that is present also in preclinical models. In this study, similar to previous reports antioxidant treatment reverted the increase in BP in CIH rats. Indeed, Moya et al. (2016) found that rats treated with the antioxidant ebselen and exposed to CIH displayed a significant decreased of the elevated BP, suggesting that CIH-induced hypertension is critically dependent on oxidative stress. In this study, to understand the cardiovascular effects of antioxidants in CIH, we assayed the *ex vivo* vascular function in two representative arteries (i.e., carotid and femoral). It is well established that NAC antioxidant effect occurs mainly by restoring the potent intracellular reducing agent glutathione, under oxidative stress conditions (Rushworth and Megson, 2014). Notably, *in vivo* NAC treatment had limited effects on the *ex vivo* impaired endothelial dependent-relaxation and increased contractile reactivity in carotid arteries, as well as partially reverted the endothelial dysfunction in femoral arteries from animals exposed to CIH. These data suggest that the antihypertensive effect of NAC and other antioxidants occurs partially by improving vascular reactivity and, in a higher degree, by preventing the overactivation of the CB in CIH. Indeed, previous reports have shown that oxidative stress in the CB drives the chemosensory potentiation and hypertensive effects of CIH (Del Rio et al., 2016; Iturriaga et al., 2017). However, considering that our studies on endothelial-dependent relaxation were carried-out after pre-constriction with KCl, a potential effect of NAC enhancing the role of endothelium-derived hyperpolarizing factor (EDHF) (Krummen et al., 2006) or alternative glutathione-dependent vasodilator pathways (Yang and Wang, 2015) cannot be ruled out.

Conversely, endothelial dysfunction has been proposed to play a key role in the cardiovascular risk associated with OSA and CIH. Indeed, OSA patients show a reduced flow-mediated dilation (Ip et al., 2004), and endothelial dysfunction has been directly demonstrated in middle cerebral (Phillips et al., 2004) and carotid arteries from rats exposed to CIH (Krause et al., 2015). In the present study, we add new evidence that vascular dysfunction in CIH is associated with an increased contractile response and impaired NOS-dependent relaxation in femoral arteries. This new data shows the effects of CIH on the vascular function on carotid and femoral arteries. In this context, we previously reported that the reduced relaxation in response to ACh in carotid arteries from CIH-treated rats can be prevented by the *ex vivo* inhibition of arginase activity, and this effect could be explained by an unbalanced endothelial expression of eNOS and arginase-1 (Krause et al., 2015). Increased arginase expression and its effect on NOS-dependent relaxation have been extensively reported in hypertension and cardiovascular diseases (Caldwell et al., 2015). For instance, a study in humans shows that plasma levels of arginase are increased in subjects with OSA that present a normal cardiovascular function compared with health controls, and these levels are further increased when OSA is associated to vascular dysfunction (Yüksel et al., 2014). In that regard, a higher arginase expression and activity has been reported in diverse models of hypoxia (i.e., intermittent and sustained) in which an increased expression of arginases 1 or 2 occurs in the endothelium leading to a decreased NO synthesis (Krotova et al., 2010; Krause et al., 2013, 2015; Singh et al., 2014; Cowburn et al., 2016). Here we found that chronic *in vivo* arginase inhibition with ABH reverted the increase in BP induced by CIH, with no effects on circulating markers of oxidative stress. Furthermore, the treatment with ABH potentiated the vasorelaxation response to ACh in carotid and femoral arteries, as well as partially reverted the increased contractile response to KCl. The later effect of ABH treatment on the contractile response could result from the inhibition of the proliferative stimulus by arginases-derived polyamines on vascular smooth muscle cells (Ignarro et al., 2001; Wei et al., 2001; Chen et al., 2009). Similar to our results, previous studies in spontaneously hypertensive rats have demonstrated the potential therapeutic role of arginase inhibitors decreasing arterial blood pressure and restoring endothelial function (Demougeot et al., 2005; Bagnost et al., 2008, 2010). Altogether, the available information reinforces the idea that arginase-mediated impaired endothelial NO synthesis plays a key role in the vascular dysfunction associated with CIH and OSA.

It is worth to note that neither NAC nor ABH treatments separately led to a complete normalization of *ex vivo* vascular responses in carotid or femoral arteries from CIH rats, despite that they had a clear effect decreasing BP, but a heterogeneous outcome on systemic oxidative stress levels. This confirms the notion that CIH-induced increase in BP (Del Rio et al., 2016; Iturriaga et al., 2017), similarly to other hypertensive models (Godo and Shimokawa, 2017; Handy and Loscalzo, 2017; Mancia et al., 2017), is a multifactorial process in which a CB chemosensory and sympathetic overactivity, oxidative stress and endothelial dysfunction are the cornerstones. Considering this idea, we aimed to determine whether the combined treatment with ABH and NAC could normalize the vascular function in CIH rats. Clearly, the combination of both drugs normalized BP and oxidative stress markers; effects accompanied by the restoration of normal *ex vivo* contractile and relaxing responses. Compared with ABH treatment, the NAC/ABH combination led to a lower, but normal, maximal NOS-dependent relaxation in carotid and femoral arteries. This counterintuitive finding could result from the buffering effect of NAC, as well as glutathione, on NO levels (Hu et al., 2006; Keszler et al., 2010; Kolesnik et al., 2013), that would be limiting the enhanced levels of NO as a consequence of arginase inhibition. Conversely, ABH/NAC combination reverted the increase in the KCl contractile response induced by CIH. Notably, in a recent study we found that an increased KCl-induced contractile response is directly associated with biomechanical and histological markers of vascular remodeling (Cañas et al., 2017). Altogether, this data strongly suggests that ABH/NAC combination reverted the vascular remodeling observed in rats exposed to CIH (Krause et al., 2015). The mechanisms involved in the combined effect of ABH-NAC, as well as, the heterogeneous changes induced in femoral and carotid arteries need further studies that would include the analysis of alternative vasodilator pathways involving the cysteine and glutathione metabolism (Yang and Wang, 2015). Nonetheless, considering the changes in ACh potency (i.e., sensitivity) and markers of vascular remodeling, this combined effect could result from an increased vascular bioavailability of NO.

Present results show that CIH increased BP in 3–5 days. This fast increase in BP is probably triggered by a potentiated sympathetic vasoconstrictor tone on the arterial blood vessels, that would result from the cyclic hypoxic excitation of the CB (Iturriaga et al., 2017). In agreement to this explanation, we found that external carotid arteries from rats submitted to CIH for 21 days showed moderate enhanced contractile responses to KCl and a diminished vasorelaxation to ACh (Krause et al., 2015). Recently, Del Rio et al. (2016) found that the ablation of both CBs completely reverts the increased BP and sympathetic overflow in hypertensive rats exposed to CIH for 21 days, indicating that the CIH-enhanced CB chemosensory drive mediates the onset and maintenance of neurogenic hypertension. It is known that CIH reduced the ACh-mediated vasodilation (Tahawi et al., 2001; Dopp et al., 2011; Krause et al., 2015). However, there are some reports showing normal endothelial function in hypertensive rats exposed to CIH (Julien et al., 2003; Lefebvre et al., 2006). Indeed, Lefebvre et al. (2006) found that the ACh-induced vasodilation in carotid, aortic and mesenteric vascular beds, as well as the contractile responses to noradrenaline and angiotensin II (Ang II), are similar between CIH-treated and sham rats, while the contraction induced by exogenous application of ET-1 is augmented in CIH-treated rats. Oxidative stress is associated with the endothelial dysfunction in CIH-treated rats. Indeed, treatment of CIH-exposed rats with allopurinol improves the reduced vasodilatation induced by ACh in gracillis arteries, although neither allopurinol nor CIH affect the vessel morphometry and systemic markers of oxidative stress in rats exposed to CIH for 14 days (Dopp et al., 2011). Similarly, Philippi et al. (2010) report that CIH elicited systemic oxidative stress, although they found that CIH has an inconsistent effect on the oxidative stress marker 3-nitrotyrosine in the vascular wall. Therefore, it is plausible that NAC and ABH may affect CIH-induced hypertension acting at different levels. It is likely that NAC may reduce the enhanced CB chemosensory discharge as all tested antioxidants do (Peng et al., 2003; Del Rio et al., 2010; Moya et al., 2016), while ABH may act at the level of the arteries. In addition, it is plausible that NAC may affect the arginase activity in the blood vessels or ABH may affect the oxidative stress in the blood vessels. If these aspects are different, it may explain the heterogeneous effect of the drugs in the femoral and carotid arteries.

## Perspectives

Present results support a potential therapeutic application of a combined antihypertensive treatment with an antioxidant and an arginase inhibitor, which not only decrease BP but also normalize endothelial vascular reactivity and revert vascular remodeling, without compromising kidney and liver functions. Further studies are required to demonstrate whether this increased antihypertensive effect is limited to the drugs tested in the present study or can be applied to other combination of antioxidant and antihypertensive agents.

## Author contributions

BK, PC, and RI conceived and designed the experiments. MP synthetized ABH. BK, ACD, PA, RI, VV, and GA performed the experiments. BK, RI, GA, VV, and ACD analyzed the data. RI and BK wrote the draft paper and all authors contributed to the manuscript and approved the final version.

### Conflict of interest statement

BK, PC, MP, and RI presented the solicitude of a patent for the pharmaceutic combination for the treatment and prevention of arterial hypertension and vascular dysfunction, number 201602951 INAPI, Chile, and PCT international protection PCT/CL2016/050062 on November 18th, 2016. The remaining authors declare that the research was conducted in the absence of any commercial or financial relationships that could be construed as a potential conflict of interest.
